# Advances in plant molecular biology: towards new challenges

**DOI:** 10.1093/jxb/erad350

**Published:** 2023-10-13

**Authors:** Angeles Aroca, Irene García

**Affiliations:** Departamento de Bioquímica Vegetal y Biología Molecular, Universidad de Sevilla, C/ Profesor García González, 1, 41012, Sevilla, Spain; Instituto de Bioquímica Vegetal y Fotosíntesis, IBVF (Universidad de Sevilla, Consejo Superior de Investigaciones Científicas), Américo Vespucio, 49, 41092, Sevilla, Spain; Instituto de Bioquímica Vegetal y Fotosíntesis, IBVF (Universidad de Sevilla, Consejo Superior de Investigaciones Científicas), Américo Vespucio, 49, 41092, Sevilla, Spain

**Keywords:** Biotic and abiotic stress, CRISPR/Cas9, plant nutrition, post-translational regulation, prime editing, transcriptional regulation

**Plant molecular biology has evolved with substantial progress in recent years, in order to decipher the molecular principles that underlie plant biology. Regulation of multiple pathways and regulatory targets for plant development, as well as adaptation to different stresses, have been elucidated during the last years, enlightening the knowledge we have about how plants cope with different environmental stimuli and how symbiosis with other organisms is used as a sustainable source of biostimulants in agriculture. The accumulated knowledge of plant molecular biology has also been enhanced due to the improvement of molecular techniques and the biotechnology of plants for gene editing. These advances have been included in this Special Issue due to the increased interest of the plant scientific community in facing significant challenges in the 21st century, in particular the need to increase global food supply under the increasing threats from climate change.**

During the last decade, trending topics in research have therefore been focused on unveiling several approaches that improve crop production, such as how to improve or trigger plant mechanisms to deal with abiotic and biotic stress; the role of hormones and how to regulate them in order to improve resistance or enhance plant development; mechanisms to improve the nutritional state in crops in order to avoid waste of chemical fertilizers; and the advancement with new technologies such as gene editing with the aim of crop improvement.

## Regulation of processes against abiotic stress

Abiotic stress triggered by different environmental conditions such as temperature, drought, light, salinity, or heavy metals has a significant impact on crop production worldwide (Kopecká *et al.,* 2023), and crop yields are expected to be reduced as a consequence of abiotic stress in the near future due to climate change and the side effects of the increased world population that force urban areas to be extended and therefore limit agriculture to areas less appropriate to crop cultivation (Döös, 2002). Abiotic stress causes >50% losses in crop yield every year, affecting plant growth, flowering, fruiting, and maturity; and reduces crop quality, including taste, nutritional composition, and market value (Oshunsanya *et al.,* 2019). Furthermore, abiotic stress weakens plants and makes them more susceptible to pests, diseases, and opportunistic pathogens. Plants employ various adaptive mechanisms to cope with abiotic stress, including altering their physiology, morphology, and biochemistry. However, prolonged or severe abiotic stress can have significant detrimental effects on plant health and crop productivity. These circumstances push researchers to find agricultural solutions against the negative impacts of abiotic stress caused by a growing population and climate change.

To mitigate the negative effects of abiotic stress on crop production, the scientific community is currently studying several approaches in depth. Under abiotic stress, one of the main plant responses is the accumulation of reactive oxygen species (ROS) (Nadarajah, 2020), reactive nitrogen species (RNS) (Wani *et al.,* 2021), and reactive sulfur species (RSS) (Alvi *et al.,* 2023), which also activate several signalling pathways. The signal transduction mediated by ROS includes a vast network which is of increasing interest in cell biology. Therefore, the redox regulation system, thioredoxins, and glutaredoxins are important players in redox homeostasis, which has been reviewed (Sevilla *et al.,* 2023), focused on their action in stress-related processes related to organellar retrograde communication and nuclear transcription. Nevertheless, further research is needed as knowledge of the signalling pathways is still limited and several players still need to be identified.

Furthermore, abiotic stress causes an important disturbance in the regulation of transport of macro- and micronutrients and heavy metals, which has been the focus of important publications in the last years. In this Special Issue, the regulation of ion channels/transporters by ROS and RNS at the transcriptional and post-translational level has been reviewed (Sandalio *et al.,* 2023). In relation to this, potassium fluxes have been demonstrated to regulate essential transpiration processes affecting the tolerance to temperature and oxidative stress, and, therefore, a review of modulation of potassium homeostasis in plants has been included in this Special Issue (Mulet *et al.,* 2023). These authors propose that potassium homeostasis is an excellent target for crop biotechnological improvement as optimizing potassium uptake, translocation, and compartmentalization would increase plant nutrition and also crop yield, and therefore it is time to apply the knowledge from the model plant Arabidopsis to crops to generate new varieties that can contribute to guaranteeing food for the increasing world population.

Besides transporters, other cellular responses, such as autophagy, are activated in plants when environmental conditions change. Autophagy is a conserved process in eukaryotic cells, where cellular components are degraded into the vacuoles in photosynthetic organisms to maintain cell homeostasis. Autophagy is induced by a wide range of conditions and its fine regulation by the transcriptional and post-translational network has been reviewed (Agbemafle *et al.,* 2023). These authors propose a coordinated regulation by transcriptional signals to replenish the proteins in the autophagy machinery and by post-translational modifications to maintain the stability and tune the function of ATG proteins.

Another important process regulated post-translationally and transcriptionally is photorespiration, which has always been considered a wasteful pathway, and research was focused on bypassing photorespiration to increase crop yield. However, in the last decade, its role in plant metabolism constitutes a hot topic, since suppressing photorespiration, although at first it in fact increases biomass, is detrimental by decreasing food quality and productivity in soils due to nitrogen and sulfur assimilation being dependent on photorespiration. Therefore, reviewing the new insights of photorespiration regulation is also a focus of this Special Issue (Aroca *et al.,* 2023) due to the fact that currently the atmospheric CO_2_ concentration is increasing and consequently the photorespiration rate is decreasing, threatening food quality and yield in the near future.

## Regulation of processes against biotic stress

Plants are constantly subjected to attack by pathogens, including bacteria, viruses, fungi, and invertebrates. Plant diseases, indeed, represent a serious loss in agriculture.

Soil-borne plant microorganisms can cause severe diseases of hundreds of agricultural crop species, causing huge yield losses of 30–90% of economically important crops such as wheat, maize, cotton, vegetables, and fruit (Bertoldo, 2014; Mihajlovic *et al.,* 2017). Invertebrates are also the origin of devastating crop losses, and the Food and Agriculture Organization (FAO) estimates that annually up to 40% of global crop production is lost to pests. Each year, plant diseases cost the global economy over US$220 billion. Globalization and global climatic change increase the occurrence of invasive pests and diseases in agricultural and forestry ecosystems, with a subsequent economic cost which is estimated, for invasive insects alone, to be at least US$70 billion.

Phytochemical control of crops disrupts the ecological balance, causes human and animal health hazards, and damage to aquatic ecosystem and beneficial organisms in the soil (Panth *et al.,* 2020). Designing alternative, sustainable, strategies is a challenge for 21st century plant biologists and, to do this, a greater understanding of plant–microbe interactions is essential.

As sessile organisms, unable to move to avoid adverse conditions, plants have extremely efficient mechanisms for detecting and responding to a wide diversity of stresses. In this Special Issue we include a review of a recently described role for a ‘traditional’ poison, hydrogen cyanide (HCN), as a signalling molecule, and specifically its function in potentiating the response of plant to biotrophic pathogens (Diaz *et al.,* 2023). The authors also review the possible mechanisms of action of this molecule, including a new post-translational modification of proteins, whose characterization is being investigated (García *et al.,* 2019).

Since they are continuously attacked by various types of pathogens, plants need to (i) identify the type of pathogen and (ii) respond by activating precise defence mechanisms. Inducible defence systems include the recognition at the membrane level of pathogen-associated molecular patterns (PAMPs) through receptor-like kinase (RLK) proteins (Shiu and Bleecker, 2001; Couto and Zipfel, 2016), and the elicitation of a resistance response (PAMP-triggered immunity, PTI), which can effectively stop the progression of infections. However, pathogens evolved and are able to bypass the plant response through the production of avirulence factors. Plants in an arms race are capable of detecting the pathogen avirulence factors, or effectors, through intracellular nucleotide-binding leucine-rich repeats proteins (NLRs), triggering effector-triggered immunity (ETI) (Peng *et al.,* 2018; Torres *et al.,* 2006). This Special Issue includes a review of the regulation of NLR defence genes through plant miRNAs including the generation of phased secondary siRNAs (López-Márquez *et al.,* 2023). The authors propose that release of miRNA/phasiNLR repression of NLR enhances NLR availability and increases plant responsiveness to pathogens, including new links between PTI and ETI. At a higher level of complexity of the pathogen and plant–host interactions in the framework of co-evolution, other authors have reviewed the pathogen effectors acting as suppressors of ETI (Rufián *et al.,* 2023). These authors discuss different modes of action for these effectors, including their target of multiple protein complexes, and their interaction with other effectors and PTI suppression. They also highlight the importance of using original pathogen systems for the design of strategies to improve crop immunity.

## Regulation of hormones

Orchestration of a plant’s response to internal and external stimuli is controlled by plant hormones (Kamiya, 2010). They regulate growth, development, and physiological processes throughout the plant’s life cycle, and their ability to fine-tune cellular response enables the plant to respond and adapt to changing environmental conditions, coordinate growth patterns, and mount defence mechanisms against any kind of stress. Each plant hormone acts independently or in combination, shaping various aspects of plant growth, development, and responses to external stimuli.

Since the discovery of the first plant hormone more than a century ago (Kögl and Kostermans, 1935), extensive research has unveiled an array of plant hormones together with their mechanisms of action (Blázquez *et al.,* 2020). As regulators of plant performance, they offer various agronomical applications to optimize crop yields and quality, acting from seed germination to fruit ripening and post-harvest preservation, including stress tolerance. It is therefore undeniable that hormone research is of great interest (Hirayama and Mochida, 2023).

Unlike animal systems, where specialized organs produce one or several hormones, plant cells are able to synthesize all plant hormones, each with unique roles and functions. Wong *et al*. (2023) review the spatial regulation of plant hormone action, delving into the contribution of the metabolism, transport, and signalling of plant hormones in their spatial specificity and action. Wong *et al.* (2023) focus on patterning processes in the plant root and shoot meristems, response to shading, and water deficit, encompassing a wide and diverse range of plant responses. They also provide a brief overview of emerging technologies such as single RNA-seq or the use of sensors that can contribute to a better understanding of the intricate action of plant hormones.

Diverse signalling molecules, including gasotransmitters, have the capacity to interact with phytohormones, enabling modification of the hormone signalling pathway and restructuring of plant responses to stimuli. Sanchez-Corrionero *et al*. (2023) review the control of the maintenance of the initial stem cell niche (SCN) cells in primary root development as well as regeneration after damage, processes that are regulated by nitric oxide (NO) and its interaction with several plant hormones. Indeed, NO interacts with the signalling pathways of auxin and cytokinin during primary root formation and regeneration, and those of brassinosteroids and cytokinin during adventitious root formation (Sanchez-Corrionero *et al.,* 2023). The authors also describe the implication of NO in root regeneration under hypoxia-related stress through the interaction with phytoglobins.

In addition, post-translational modifications of proteins regulate phytohormone signalling. This is the case of protein *O*-glycosylation, reviewed in this issue by Xu *et al.* (2023). The authors describe the different types of *O*-glycosylation which exist in plants, as well as the methods to identify them. Their function in cell wall, flower, and fruit development is also discussed together with their interaction with phytohormones. Interaction between the *O*-fucosyltransferase SPY and several hormone signalling pathways is described, such as negative interactions with gibberellin, abscisic acid, and brassinolide signalling by enhancing DELLA activity by *O*-fucosylation. SPY also interferes with the signalling pathways of jasmonate and abscisic acid, facilitates the ethylene response to ripening, and enhances the cytokinin response.

Finally, in a research paper, Borah *et al*. (2023) investigate the molecular mechanisms of a very specific process, the lignification of anthers in rice, in response to jasmonic acid. In the presence of jasmonic acid, the molecular complex between the E3 ubiquitin ligase OsATL53 and its degradation target, the cinnamoyl CoA-reductase OsCCR14, is located in the nuclear periphery. This perinuclear location induces the activity of the F-box protein OsFBK1, that degrades OsATL53 and therefore increases the activity of OsCCR14 and the lignification process.

In summary, this issue provides a comprehensive understanding of plant hormones as crucial molecular regulators, including spatial regulation, interactions with other signalling molecules, and molecular mechanisms of action, and inspires further research to unlock the full potential of plant hormone manipulation for sustainable agriculture and global food security.

## Improvement of plant nutrition

Plants need nutrients for growth and development, and the nutritional state of plants directly affects food quality, so optimizing plant nutrition has always been a focus of plant research. Several agricultural practices have historically been developed to improve plant nutrition in order to optimize crop yield, such as fertilization, rotation, and irrigation, but scarceness of micronutrients has a wide impact on plants and, therefore, on human and animal health.

Ammonium fertilizers are the main and most popular farming practices worldwide, helping farmers to produce higher yields to feed a growing world population. However, it is well known that nitrogen excess negatively impacts air pollution, acid rain, and aquatic ecosystems (Wyer *et al.,* 2022). In addition, ammonium uptake and assimilation also affect the availability of other macro- and micronutrients. Therefore, innovative farming practices that include sustainable crop breeding are needed. In this Special Issue, the current knowledge of the interaction between ammonium and other essential mineral elements together with soil pH has been reviewed to help a better understanding of fertilizer formulas that would improve crop productivity (Coleto *et al.,* 2023). In relation to this need for sustainable agriculture, another challenge is the use of bio-fertilizers to address the toxicity of the chemical N fertilizers. Microbial inoculations are used for bio-fortification, as bio-fertilizers and bio-pesticides, which, in contrast to chemical fertilizers, have no damaging effects on the ecosystem (Maitra *et al.,* 2021). In this Special Issue, the symbiosis between cyanobacteria and plants has been reviewed (Álvarez *et al.,* 2023), deciphering the molecular mechanisms involved in this crosstalk, that will help to further design strategies for bio-inoculant formulations.

## New technologies: gene editing

Gene editing in plants has emerged as a powerful tool for crop improvement and agricultural sustainability. Using genetic manipulation, desirable traits in plants can be enhanced, such as disease resistance, nutritional content, yield, and stress tolerance (Abdul Aziz *et al.,* 2022; Esmaeili *et al.,* 2022). Therefore, gene editing technologies have significantly accelerated plant research and breeding efforts, addressing agricultural challenges, improving crop productivity, and contributing to sustainable and resilient food systems. The most well-known and widely used gene editing technology is CRISPR/Cas9 [clustered regularly interspaced palindromic repeats (CRISPR)/CRISPR-associated protein 9]; however, beyond this technique, other gene editing technologies also exist, such as zinc finger nucleases (ZFNs), transcription activator-like effector nucleases (TALENs), and homing endonucleases (HEs) (Khalil, 2020; Abdallah *et al.,* 2021). Although editing of coding genes has significantly progressed in recent years, studies on the editing of non-coding regulatory DNAs are still limited. However, the use of CRISPR/Cas9 together with non-coding regulatory DNAs would provide a promising tool for crop breeding. In this Special Issue, these gene editing technologies have been reviewed and possible solutions to existing problems discussed (Chen *et al.,* 2023). Likewise, a prime editing approach is a modified CRISPR/Cas9 approach that offers enhanced precision and versatility by introducing modifications in the genome compared with traditional gene editing techniques (Huang and Liu, 2023). Prime editing has shown improved efficiency compared with other gene editing methods, reducing the likelihood of incomplete or inefficient modifications (Li *et al.,* 2021; Ahmad *et al.,* 2023). A description of an improved technology has been included in this issue to describe prime editing in the model plant *Physcomitrella patens*, editing for the first time two independently coded peptides (Perroud *et al.,* 2023).

## Concluding remarks

The articles presented in this issue encompass a wide range of topics within the different areas of knowledge related to plant biology that address present and future threats. Throughout their life cycle, from seed to maturity, plants encounter internal and external stimuli that influence their fitness and production. Knowledge of proper plant nutrition and its ability to adapt to environmental changes and cope with biotic and abiotic stresses, along with their consequences, is essential to achieve optimal crop production. Multidisciplinary research focused on crop production is being quickly generated to overcome the needs of a growing population, and new challenges are needed to cope with climatic change (Box 1). Sharing research advancements with the international scientific community has accelerated the progress of knowledge in all fields. The biannual Plant Molecular Biology Meeting, which was the impetus for this Special Issue, is a great example of a scientific meeting where molecular plant biologists discuss new techniques and strategies to improve crop production. Therefore, this Special Issue unveils new approaches and solutions to inspire further research and scientific collaborations to address the urgent need to improve plant productivity under current global environmental changes.

**Box 1. New challenges for plant biologists in a scenario of climate change**

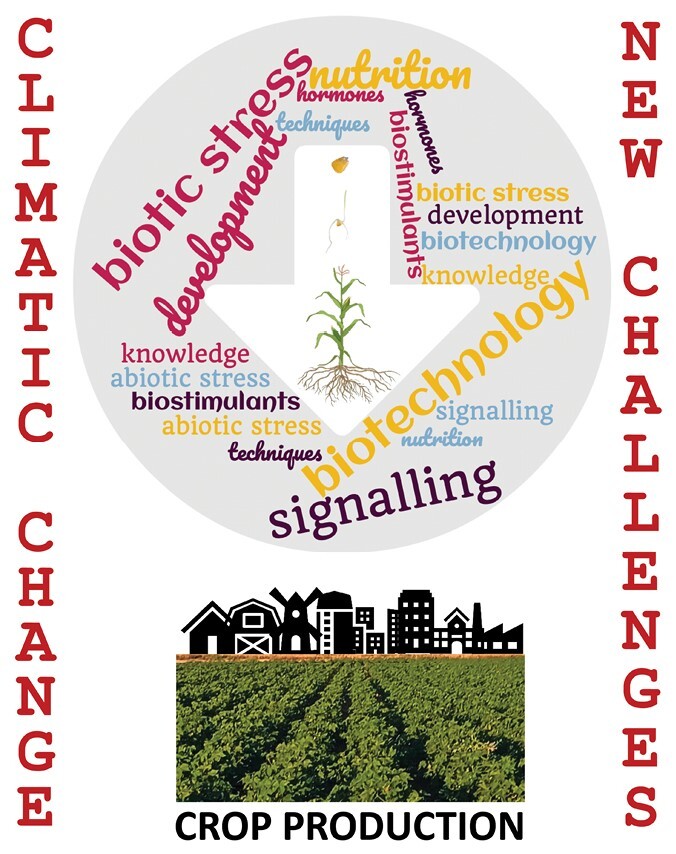

Crop production requires sustainable improvements to meet the food and raw material demands of a growing population while safeguarding ecosystems. Plant biologists play an important role in this task by generating knowledge and improving new techniques and strategies to manipulate plant fitness and production under both normal and adverse or varying conditions. Part of the figure was created with BioRender.com.
